# Parkinson’s Disease Non-Motor Subtypes Classification in a Group of Slovenian Patients: Actuarial vs. Data-Driven Approach

**DOI:** 10.3390/jcm12237434

**Published:** 2023-11-30

**Authors:** Timotej Petrijan, Jan Zmazek, Marija Menih

**Affiliations:** 1 Department of Neurology, University Medical Center Maribor, 2000 Maribor, Slovenia; marija.menih@gmail.com; 2 Department of Physics, University of Maribor, 2000 Maribor, Slovenia; jan.zmazek@um.si

**Keywords:** Parkinson’s disease, non-motor symptoms subtypes, a priori classification, cluster analysis

## Abstract

Background and purpose: The aim of this study was to examine the risk factors, prodromal symptoms, non-motor symptoms (NMS), and motor symptoms (MS) in different Parkinson’s disease (PD) non-motor subtypes, classified using newly established criteria and a data-driven approach. Methods: A total of 168 patients with idiopathic PD underwent comprehensive NMS and MS examinations. NMS were assessed by the Non-Motor Symptom Scale (NMSS), Montreal Cognitive Assessment (MoCA), Hamilton Depression Scale (HAM-D), Hamilton Anxiety Rating Scale (HAM-A), REM Sleep Behavior Disorder Screening Questionnaire (RBDSQ), Epworth Sleepiness Scale (ESS), Starkstein Apathy Scale (SAS) and Fatigue Severity Scale (FSS). Motor subtypes were classified based on Stebbins’ method. Patients were classified into groups of three NMS subtypes (cortical, limbic, and brainstem) based on the newly designed inclusion criteria. Further, data-driven clustering was performed as an alternative, statistical learning-based classification approach. The two classification approaches were compared for consistency. Results: We identified 38 (22.6%) patients with the cortical subtype, 48 (28.6%) with the limbic, and 82 (48.8%) patients with the brainstem NMS PD subtype. Using a data-driven approach, we identified five different clusters. Three corresponded to the cortical, limbic, and brainstem subtypes, while the two additional clusters may have represented patients with early and advanced PD. Pearson chi-square test of independence revealed that a priori classification and cluster membership were significantly related to one another with a large effect size (χ^2^(8) = 175.001, *p* < 0.001, Cramer’s V = 0.722). The demographic and clinical profiles differed between NMS subtypes and clusters. Conclusion: Using the actuarial and clustering approach, marked differences between individual NMS subtypes were found. The newly established criteria have potential as a simplified tool for future clinical research of NMS subtypes of Parkinson’s disease.

## 1. Introduction

Parkinson’s disease (PD) is the second most common neurodegenerative disease, affecting over 3% of people over 70. Until recently, the classic view of PD prevailed, which is that PD is primarily a movement disorder, resulting from affected dopaminergic neurons of the substantia nigra. The diagnosis is based on clinical criteria that require the presence of motor symptoms and signs (MS), but do not include non-motor symptoms and signs (NMS). However, the NMS are an important part of the clinical spectrum of PD and appear many years before MS in a significant proportion of patients [1].

Neuroanatomically, NMS are divided into symptoms of the cerebral cortex, basal ganglia, brainstem, peripheral nervous system, etc. [2]. Most result from the neurodegenerative process. NMS can develop in all stages of the disease. Rapid eye movement sleep behavior disorder (RBD), constipation, depression, and olfactory disturbances can appear 20 or more years before MS [3]. This leads to the development of the concept of preclinical/asymptomatic and prodromal/premotor PD. Braak et al. [4] described the α-synuclein and Lewy body deposition as a key pathophysiological mechanism of PD, starting with the caudal brainstem and olfactory bulb, followed by spread to the mesencephalon, limbic cortex, and neocortex. This concept has been expanded through studies examining the involvement of the autonomic nervous system [4]. Further, the hypothesis of an extra-nigral and peripheral onset of the disease initially causing NMS has been confirmed by numerous clinical studies [5].

A recent review highlighted the importance of including NMS in subtyping, traditionally dominated by motor symptoms [6], and may not simply reflect different stages of disease progression [7]. So far, a few NMS-dominant subtypes of PB have been described in the literature; the cognitive subtype [8], a subtype of apathy-predominant patients [9], a depression/anxiety subtype [10], the subtype with predominant sleep disorders [11], the pain-dominant subtype [8], subtypes with predominant fatigue, autonomic nervous system impairment, and weight loss [8]. Clinical subtyping based on NMS is a relatively new and poorly researched concept. On the basis of previously established hypotheses, patients are divided into subtypes, also termed a priori classification approach. Actuarial classification criteria are used, defined as using objective, pre-established numerical definitions of impairment of interest. On the other hand, the clustering analysis algorithm divides patients into groups based on the similarity or co-occurrence of certain characteristics included in the analysis [12]. Once the subtypes are identified, their differences are sought [13].

A well-known actuarial classification is from 1990 by Jankovic et al. [14]. Based on the DATATOP study, they defined two groups of PD patients based on clinical characteristics of MS (tremor and PIGD subtype). The criteria for the different groupings were determined before the data analysis and were based on previously reported possible subtypes and the collective clinical experience of the investigators. They used The Unified Parkinson’s Disease Rating Scale, calculating an average global tremor and PIGD scores [15]. Comparisons of the groups provided support for the existence of clinical subtypes. In 2013, Stebbins et al. [16] provided a new empiric method using a new scale, namely, the MDS-UPDRS [17]. They developed comparable and valid PIGD and TD scores. Following the example of the studies mentioned above, we formulated criteria for classifying patients into subtypes based on the Non-Motor Symptom Scale (NMSS). Cortical, limbic, and brainstem subtypes were defined based on previously reported possible subtypes.

The most common clustering techniques are hierarchical and partitioning [18]. In partitioning cluster analysis, data are divided into non-overlapping subsets where each data instance is assigned to precisely one subset [19]. K-means clustering is one of the simplest methods [19]. According to a recent systematic review of PD cluster analysis research, 13 studies utilized K-means cluster analysis, with a series of studies evaluating two to five subtypes [18].

Recently, the actuarial and clustering classification approaches were compared in patients with mild cognitive impairment (MCI) in Alzheimer’s disease (AD) and in PD. The actuarial approach produced reliable cognitive subtypes in AD, and data-driven algorithms enhanced diagnostic sensitivity relative to consensus diagnosis for identifying older adults at risk for cognitive decline [20]. Kenney et al. [21] highlighted the utility of comparing actuarial and data-driven approaches to establish concurrent validity of cognitive impairment in PD. They mapped actuarial criteria for MCI in PD onto data-driven cognitive subtypes. However, no studies thus far reported a comparison of these classification approaches in PD subtyping based on NMS.

So far, many risk factors for the development of various NMS have been identified; age, gender, disease duration, level of education, severity of motor symptoms and signs, disease stage, higher doses of levodopa, depression, apathy, excessive daytime sleepiness, cognitive decline, among others [1]. Risk factors for different NMS often overlap, suggesting a possible existence of different NMS subtypes. Sociodemographic variables have emerged as significant risk factors for individual NMS [1]. The female gender was associated with more frequent hallucinations, depression, and insomnia, and the male gender was associated with earlier cognitive decline, which is specific to PD [22]. Older age is a risk factor for dementia, apathy, and hallucinations [23], while lower education is a risk factor for cognitive decline, dementia, and apathy [1]. On the other hand, younger patients had a higher risk of developing depression [22]. Functional, neurochemical, and structural asymmetry or lateralization of the brain is a well-explored concept, particularly evident in PD. However, the mechanisms involved in the development of asymmetry are not fully understood. One possible factor is handedness [24]. Cubo et al. [25] observed a mild but significantly higher motor and overall NMS burden in patients with predominant left motor impairment. The onset of MS and handedness could potentially have prognostic value for the development of NMS.

The clinical expression of many NMS suggests that the different subtypes result from different levels of Lewy body deposition, and, thus neurodegeneration in different regions of the central and peripheral nervous system [26]. Previous hypotheses [27,28,29,30] classified the pathophysiological process as predominantly limbic, cortical, and brainstem. Sauerbiereva et al. [8] theoretically proposed a division into brainstem, limbic, and cortical NMS subtypes.

To address the lack of research into understanding the NMS-based subtyping, the aim of our study was to examine risk factors, prodromal symptoms, non-motor symptoms, and motor subtypes in different NMS subtypes, using newly designed actuarial and data-driven approaches.

## 2. Materials and Methods

Patients diagnosed with idiopathic PD, who were examined at the University Department of Neurology, University Medical Centre Maribor between 2013 and 2023, were recruited for an ethically approved research study (Slovenian National Medical Ethics Committee, No. 0120-509/2019/4).

Inclusion criteria were diagnosed idiopathic PD based on UK PD Society Brain Bank Diagnostic Criteria [31], age ≥ 18 years, Hoehn and Yahr (H&Y) 1–5. The exclusion criteria were:

Presence of atypical and secondary parkinsonism, presence of significant cognitive impairment (MoCA < 20), and presence of major psychiatric disturbance.

### 2.1. Clinical Assessments

Demographic and clinical characteristics were collected from patients. These included gender, education (according to the International Standard Classification of Education—ISCED), handedness, age at assessment, age at onset, disease duration (<5 years, 5–10 years, >10 years), data on exposure to risk factors for the development of PD, data on the prevalence of individual NMS, first NMS and prodromal NMS.

The data were collected according to the MDS (Movement Disorder Society) Research Criteria [32]. Risk factors demonstrated in at least two prospective cohort studies or meta-analyses have been included. These are as follows: male gender, regular occupational exposure to pesticides or very frequent (≥100×) non-occupational exposure to pesticides, occupational exposure to solvents, non-consumption of caffeine (<3 cups of coffee per week), non-smoking status, and family burden. We additionally collected data on risk factors for which research findings are contradictory. These are alcohol consumption, head injury, and living in a rural environment [32].

We included the following prodromal/pre-motor NMS: RBD, smell disorder, constipation requiring treatment more than 1× per week or bowel emptying <1× every two days, excessive daytime sleepiness, symptomatic hypotension not caused by drugs, sexual dysfunction, micturition disorders except for of long-term (>10 years lasting) stress urinary incontinence in women, doctor-diagnosed depression with/without anxiety.

A neurologist with expertise in extrapyramidal disorders clinically examined the patients. Individual symptoms and signs were evaluated according to rating scales and questionnaires that were validated in the group of patients with PD and were recommended by the MDS working group.

To assess NMS, we used NMSS (Non-motor Symptoms Assessment Scale for Parkinson’s Disease), which is a validated screening tool for PD non-motor symptoms [33].

We additionally used MoCA (Montreal Cognitive Assessment) [34], HAM-D (Hamilton Depression Scale) [35], HAM-A (Hamilton Anxiety Rating Scale) [36], RBDSQ (REM Sleep Behavior Disorder Screening Questionnaire) [37], ESS (Epworth Sleepiness Scale) [37], FSS (Fatigue Severity Scale) [37], SAS (Starkstein Apathy Scale) [38].

We used the MDS-UPDRSIII (Movement Disorder Society Unified Parkinson’s Disease Rating Scale-III) scale to assess motor disability, and the H&Y scale to assess disease progression [17]. All patients were tested in the “on” phase. According to the method of Tomlinson et al. [39] we calculated the L-dopa equivalent daily dose.

Motor subtyping was based on Stebbins’ method using MDS-UPDRS [16]. Only NMS that lasted continuously for at least three months were considered. The patient’s relative or guardian was present during the structured interview for greater reliability.

### 2.2. NMS Subtyping

#### 2.2.1. A Priori Classification Approach

Depending on the predominant NMS, we divided the patients into groups of three NMS subtypes, according to the newly designed inclusion criteria (formula) described below:

1. Cortical subtype; the sum of the NMSS symptom scores in the domains of cognitive impairment and apathy (NMSS items 7, 8, 16, 17, 18) is higher than the sum of the scores in the symptom domains classified as limbic and brainstem.
2. Limbic subtype; the sum of the NMSS symptom scores in the depression, anxiety, pain, and fatigue domains (NMSS items 4, 9, 10, 11, 12, 27, 29) is higher than the sum of the scores in the symptom domains classified as cortical and brainstem.
3. Brainstem subtype; the sum of the NMSS symptom scores in the domain of brainstem symptoms (NMSS items 1, 2, 3, 5, 19, 20, 21, 22, 23, 24, 25, 26) is higher than the sum of the scores in the symptom domains classified as cortical and limbic.

#### 2.2.2. NMS Subtyping Based on K-Means Clustering

We identified data-driven NMS subtypes using the k-means clustering analysis in this same clinical cohort. The number of variables was reduced prior to clustering through principal component analysis (PCA). We determined how well NMS subtypes map onto the cluster-derived subtypes.

### 2.3. Statistical Analysis

Statistical analysis was performed using Jamovi v2.3 open statistical software and SciPy Python library v1.11. The Shapiro–Wilk test was used to check the normality of data distribution. According to the results of the normality of the data distribution, we selected appropriate parametric or non-parametric statistical tests. We rejected the null hypothesis with a 5% risk. A statistically significant difference was considered at a value of *p* ≤ 0.05. To control the false discovery rate (FDR) in multiple hypothesis testing, the Benjamini–Hochberg method was used. The FDR in Benjamini–Hochberg method was set to 0.05.

Demographic and clinical characteristic variables were first compared by their mean or proportions between the three non-motor subtypes using ANOVA, Kruskal–Wallis, or chi-squared test, depending on the types of variables. For post hoc analysis following Kruskal–Wallis tests, the Dwass–Steel–Critchlow–Fligner method was utilized for pairwise comparisons. Similarly, for the chi-squared tests of independence, post hoc testing was conducted using z scores of adjusted standardized residuals. All *p*-values from post hoc analyses were adjusted with Bonferroni corrections.

We used cluster analysis to identify homogeneous patient subgroups. This operation was carried out using the k-means algorithm. The variables considered were a subset of the most informative variables selected from the set of variables by PCA. The selected variables were standardized prior to performing cluster analysis, and the number of clusters was visually determined using a scree plot. The optimal number of clusters was determined using the elbow method. PCA and k-means clustering algorithms were implemented using the sci-kit-learn machine-learning library for Python. Demographic and clinical characteristic variables were compared by their mean or proportions between the five clusters using the Kruskal–Wallis test. To quantify the relationship between a priori classification and cluster membership, we used the Pearson chi-square test of independence.

## 3. Results

### 3.1. Demographic and Clinical Characteristics

The database search identified 300 individuals meeting inclusion and exclusion criteria. Of 300 patients, 204 were willing to participate and provided written informed consent. The analysis included 168 patients who completed the baseline MS and NMS assessments. The demographic and clinical characteristics of the overall patients with PD and the three different NMS subtypes are presented in Table 1. Of the participants, 59.9% were male, the mean age was 71.7 years, with a mean age at onset of 65.45 years. In 61.3% of patients, the diagnosis was made between 50 and 70 years of age. The disease duration was less than five years in 45.2% of patients and less than ten years in 79.8% of patients. Of the included patients, 85.7% were right-handed. For education, 73.2% of the patients had completed at least secondary school (level 3 and higher according to ISCED). Family burden was present in 16.1% of patients. We identified 87 (51.8%) patients with tremor-dominant (TD) subtype, 61 (36.3%) with Postural Instability Gait Disorder (PIGD) subtype and 20 (11.9%) with Intermediate subtype (I).

### 3.2. A Priori Classification Approach

**Figure 1:**
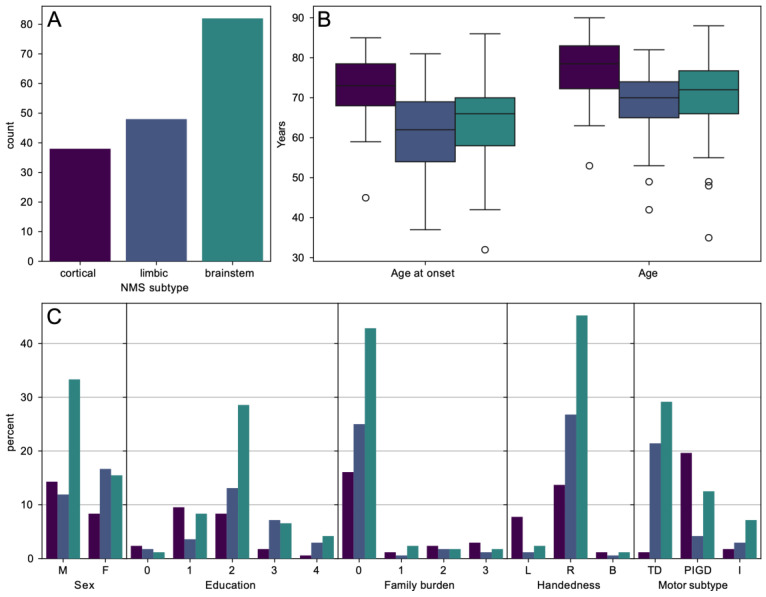
A graphical representation of the demographic and clinical characteristics of patients participating in the study, grouped by their NMS subtypes, i.e., cortical (purple), limbic (dark blue) and brainstem (green) (**A**) A number of patients with cortical, limbic, and brainstem NMS subtypes. (**B**) Boxplots of age at onset of the disease and age of patients. Errobars represent 95% confidence interval and circles represent outliers. (**C**) Distribution of sex, education, family burden (0: none; 1: sibling who had PD onset at <50 years age; 2: sibling with >50 age of onset, parent; 3: any other first-degree relative with PD), handedness, and motor subtype (TD: tremor dominant; PIGD: postural instability gait disorder; I: intermediate) by NMS subtype.

Based on the NMSS tool, the cohort was classified into three subtypes as follows: 38 (22.6%) patients presented with the cortical subtype, 48 (28.6%) patients had the limbic subtype, and 82 (48.8%) patients exhibited the brainstem subtype, as presented in Figure 1.

Across the NMS subtypes based on the a priori classification approach, the demographic and clinical profiles differed in gender (*p* = 0.02), age at evaluation (*p* = 0.055), and at onset (*p* = 0.014), education (*p* = 0.028), and handedness (*p* = 0.018) (Table 1). Further, the NMS subtypes differed in the following risk factors: alcohol (*p* = 0.006) and caffeine (*p* = 0.012) consumption, and smoking (*p* = 0.008) (Table S1). Among prodromi, smell disorder (*p* = 0.039), constipation (*p* = 0.019), and depression (*p* = 0.011) were significantly different across the NMS subtypes (Table S1). When observed at the individual level, the following NMS differences were found: smell disorder (*p* = 0.018), constipation (*p* = 0.009), micturition dysfunction (*p* = 0.01), sweating (*p* = 0.012), diplopia (*p* = 0.044), pain (*p* = 0.006), depression (*p* = 0.008), anxiety (*p* = 0.007), cognitive disorder (*p* = 0.006), and apathy (*p* = 0.006) (Table S2). We found statistically significant differences in the following rating scales and questionnaire: MoCA (*p* = 0.005), HAM-A (*p* = 0.005), HAM-D (*p* = 0.004), H&Y (*p* = 0.004), ESS (*p* = 0.006), FSS (*p* = 0.044), RBDSQ (*p* = 0.018), and SAS (*p* = 0.003). We found statistically significant differences in the prevalence of motor subtypes (*p* = 0.004) (Table 1).

### 3.3. NMS Subtypes Resulting from k-Means Clustering

**Figure 2:**
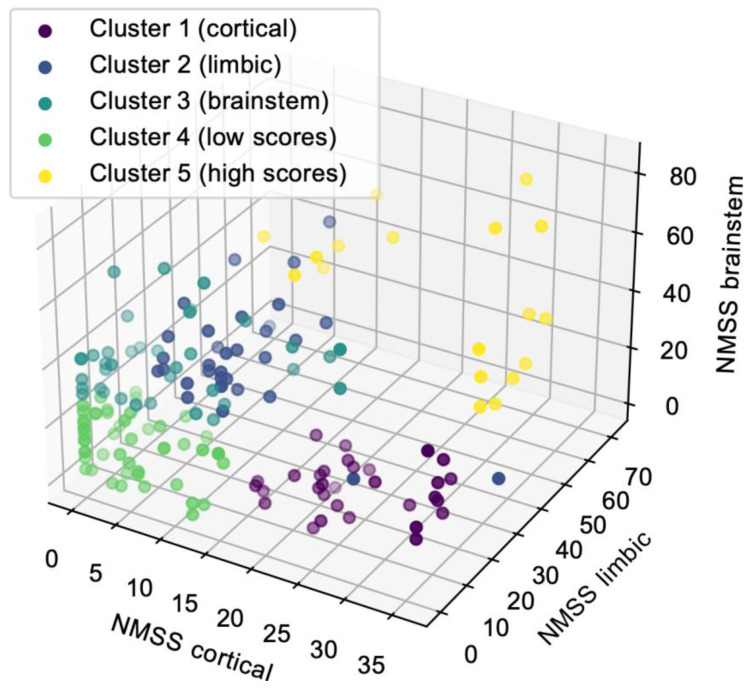
A 3D plot of the scores (NMSS cortical, NMSS limbic, and NMSS brainstem) of patients and their classification into five clusters using k-means clustering algorithm.

Classification of cohorts based on the k-means clustering identified five NMS subtypes. Visual inspection of the cluster centers (Figure 2) revealed a group of patients (cluster 1) with high scores in cortical domains and low scores in limbic and brainstem domains that correspond to the cortical subtype (n = 37, purple spots). The second group of patients (cluster 2) had high scores in limbic domains and low scores in cortical and brainstem domains that correspond to the limbic subtype (n = 35, dark blue). The third group of patients (cluster 3) had high scores in brainstem domains and low scores in cortical and limbic domains that correspond to the brainstem subtype (n = 38, turquoise spots). We additionally identified two groups of patients. The fourth group of patients (cluster 4) had low scores in each domain and could represent patients with early PD (n = 46, green spots). In cluster 4, 90.4% of patients had a disease duration <10 years (Table 2). The fifth group of patients (cluster 5) had high scores in each domain and could represent patients with advanced PD (n = 12, yellow spots). In cluster 5, 44.4% of patients had disease duration >10 years (Table 2).

Between clusters, the demographic and clinical profiles differed in gender (*p* = 0.025), age at evaluation (*p* = 0.038) and at onset (*p* = 0.019), education (*p* = 0.008), handedness (*p* = 0.004), and disease duration (*p* = 0.006) (Table 2). We found statistically significant differences between clusters in the prevalence of the following risk factors: head injury (*p* = 0.01), caffeine (*p* = 0.024), and alcohol (*p* = 0.03) consumption (Table S4). We found statistically significant differences in the prevalence of the following prodromes: constipation (*p* = 0.008), and depression (*p* = 0.006) (Table S4). We found statistically significant differences in the following rating scales and questionnaire: MoCA (*p* = 0.004), HAM-A (*p* = 0.004), HAM-D (*p* = 0.003), UPDRSIII (*p* = 0.003), H&Y (*p* = 0.003), ESS (*p* = 0.003), FSS (*p* = 0.002), RBDSQ (*p* = 0.018), SAS (*p* = 0.002), and NMSS (*p* = 0.005) (Table 2). We found statistically significant differences between clusters in the prevalence of motor subtypes (*p* = 0.003) (Table 2).

### 3.4. Relationship between NMS Subtyping Based on a Priori Approach and k-Means Clustering

**Figure 3:**
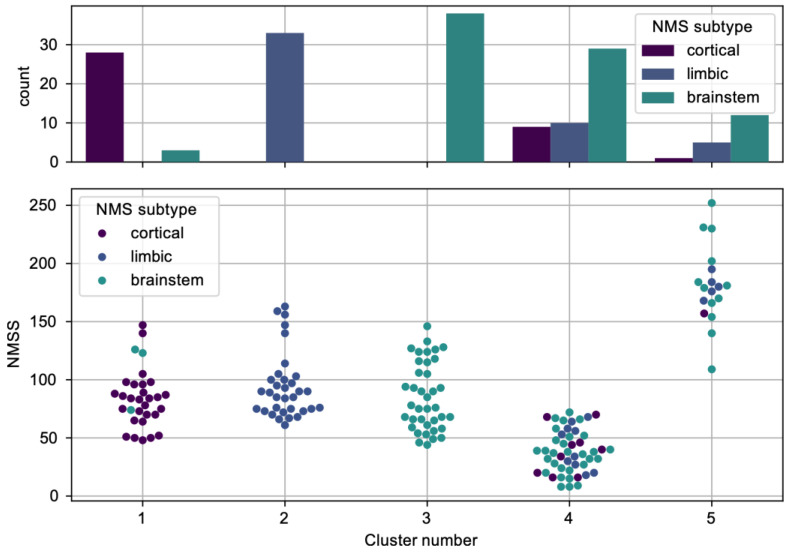
Relationship between a priori classification of NMS subtypes and cluster number. Upper panel depicts distribution (counts) of NMS subtypes for each cluster. Lower panel illustrates NMSS scores in each cluster.

To evaluate the relationship between the two classification approaches, the Pearson chi-square test of independence was applied, which identified a significant effect size: (χ^2^(8) = 175.001, *p* < 0.001, Cramer’s V = 0.722). This significant effect is additionally demonstrated in Figure 3, depicting the allocation of a-priori-based NMS subtypes within clusters resulting from k-means clustering analysis: where clusters 1, 2, and 3 correspond to cortical, brainstem, and limbic, respectively, clusters 4 and 5 indicate further subtyping, as suggested above.

## 4. Discussion

The clinical expression of many NMS suggests that the different subtypes result from different levels of Lewy body deposition and thus neurodegeneration in different regions of the central and peripheral nervous system [26]. In addition to the dopaminergic system, other neurotransmitter systems are affected [28]. Non-dopaminergic brainstem areas may be affected before dopaminergic ones [26]. Hypotheses describe the spread of the neurodegenerative process to the limbic system and brainstem area; either via the olfactory system or via the enteric nervous system and the vagal nerve [8]. Different hypotheses classify the pathophysiological process as predominantly limbic, cortical, and brainstem [27,28,29,30]. Based on this, Sauerbiereva et al. [8] proposed a division into cortical, limbic, and brainstem NMS subtypes. NMS subtyping is therefore based on the assumptions that the early and essential loss of neurons, which is crucial to the formation of NMS, begins in many non-dopaminergic nuclei of the limbic system and brainstem, either before or simultaneously with the loss of dopaminergic neurons, which is, however, key to the formation of MS [8].

Both, the actuarial/clinical theoretical as well as the clustering-analysis classification approaches aim to characterize distinct clinical subtypes in PD [21]. Several clinical scales are utilized to evaluate PD patients, but many are ordinal in type, not providing a quantifiable severity level [18]. To classify patients, we used the Non-Motor Symptoms Scale (NMSS), a 30-item rater-based scale that measures the severity and frequency of non-motor symptoms across nine dimensions [33]. These two values provide more information and a way for better separation of patients into subtypes [18].

Similar patient subtypes were noted in the clustering studies, including old age-at-onset and rapid disease progression and young age-at-onset and slow disease progression, with the addition of minor, intermediate, and severe, motor and non-motor symptom subtypes [40]. Studies used different datasets and K values based on a range of interests or past studies not specified or specific to the dataset under review [18].

Kenney et al. [21] recently investigated different techniques of methodologically defining and characterizing cognitive impairment in a large clinical sample of individuals with idiopathic PD without dementia. They also took two approaches (i.e., actuarial PD-MCI classification and cluster analytics) and looked at their overlap. They learned which cognitive phenotypes empirically emerged. Using both approaches in our study, we found apparent differences between individual NMS subtypes in the demographic and clinical profiles. A priori classification and cluster membership were significantly related to one another with a large effect size. Cluster analysis additionally identified two groups of patients. One (cluster 4) had low scores in each domain and could represent patients with early PD; the second (cluster 5) had high scores in each domain and could represent patients with advanced PD. The sample of patients in cluster 5 was, however, very small. We hypothesize that in the early stage of PD when the burden of NMS is relatively low, and in the advanced stage of the disease when the burden of NMS is rather high and there is likely to be an overlapping of subtypes, it is more challenging to classify patients into NMS subtypes.

Based on previous studies, the cognitive subtype is characterized by impairment of cognitive functions, even in the initial phase of the disease [8]. Early dementia is most likely a reflection of a significant burden of Lewy bodies in the brain cortex [41]. Williams-Gray et al. [42] observed that in this group of patients, the main characteristics were higher age (≥72 years), non-tremor-dominant motor subtype, UPDRSIII score ≥25, a low score in semantic fluency and a lower score in pentagon copying. Cognitive impairment and apathy were hallmarks of the cortical subtype in our study. The cortical subtype was more common in men, age at onset was higher than in limbic and brainstem subtypes, and more patients had the PIGD motor subtype. Patients with the cortical subtype had the lowest MoCA and the highest H&Y and SAS scores. Patients were less frequently right-handed, compared to the limbic and brainstem subtypes. This was an exciting finding. There is a relationship between handedness and AD. Some studies suggest that left-handedness or some associated factors may contribute to the early appearance of cognitive deficits during the development of AD [43]. One study reported a higher incidence of left-handedness in early-onset relative to late-onset AD cases [44]. Moreover, genetics play a significant role in determining handedness, and the same genes governing which hand you prefer are also implicated in PD and AD. The study also indicated an association between the aspects of brain development linked to handedness and the likelihood of developing PD [45]. In our study, the cortical subtype was also associated with a lower level of education. There were more smokers than in the limbic and brainstem subtypes. The most common first NMS were olfactory disorders.

A common method of empirical phenotyping is also subtyping based on age at disease onset [13]. According to various studies, early-onset PD has a slower progression [46] and less cognitive decline [47]. In our study, the brainstem and the limbic subtypes had lower average age at onset compared with the cortical subtype. The cortical subtype, on the other hand, had the highest H&Y score.

A depression/anxiety subtype can occur in early-onset or late-onset PD [10]. The pain-dominant subtype is characterized by different pain syndromes. The patients have a higher risk of developing disproportionate pain relative to disease progression and motor impairment [8]. Depression, anxiety, and pain were hallmarks of the limbic NMS subtype in our study. The limbic subtype was more common in women; the patients were younger. The limbic subtype was associated with a higher education level than the cortical and brainstem subtypes; more patients regularly consumed caffeine products. Among the most common prodromal NMS, the limbic subtype had significantly fewer smell disorders and a significantly higher depression rate. Among all NMS, pain, depression, and anxiety were found more often than in cortical and brainstem subtypes. The most common first NMS was depression. More patients with the limbic subtype had TD motor subtype, compared to the cortical and brainstem subtype. Patients with the limbic subtype had the highest HAM-A, HAM-D, FSS, and NMSS scores. However, the difference in NMSS score was not statistically significant.

Subtypes with predominant autonomic nervous system impairment and weight loss have also been identified [8]. Constipation, micturition dysfunction, and excessive sweating were hallmarks of the brainstem subtype in our study. The brainstem subtype was more common in men, and age at onset was lower than in the cortical subtype. More patients regularly consumed alcohol products compared to cortical and limbic subtypes. Among the most common PD prodromes, the brainstem subtype had a significantly higher frequency of constipation. Among all NMS, constipation, micturition dysfunction, excessive sweating, and diplopia were reported more often than in cortical and limbic subtypes. The most common first NMS was constipation; more patients had intermediate motor subtypes. Patients with the brainstem subtype had the highest ESS and RBDSQ scores.

Of patients with PD, 98.6% have NMS; the average number of NMS in an individual patient is 7.8. The number increases with the duration and progression of the disease [48]. In our study, all patients (100%) reported at least one NMS, the average number of NMS in an individual patient was 6.9. Limbic and brainstem subtypes had a higher average number of NMS in particular patients compared with cortical; however, the difference was not statistically significant. In the overall study population, we found a progressive increase in the average number of NMS within different disease duration groups.

Reported rates of smell impairment in PD patients range from 75 to 95% compared with 25% in the normal population [49]. In our study, 32.1% of PD patients reported smell disorder. It was one of the prodromal NMS in 27.4% of patients and the first NMS in 19.0%. It was curiously less frequent in the limbic NMS subtype, even though the olfactory and limbic systems are anatomically and functionally closely related. This could be explained by the higher proportion of smokers in the cortical subtype compared to the limbic and brainstem subtypes. Tobacco has also been proven to be an essential risk factor in developing olfactory disorders in the general population [50,51]. On the other hand, Sharer et al. [52] recently concluded that, differently from the general population, smokers among PD patients have less decline in their olfactory function when compared to those who do not smoke. To our knowledge, olfactory disturbances have also not been identified as an independent risk factor for developing psychiatric disorders in Parkinson’s disease [53]. On the other hand, among PD patients, anosmia has been associated with worse performance on cognitive tests and may be a predictor of emergent PD-related dementia. A longitudinal study by Baba et al. [54] identified severe olfactory dysfunction as a prodromal symptom of dementia associated with Parkinson’s disease. Interestingly, smell impairment in PD has been linked to impairment of cholinergic transmission. These data align with the observation that hyposmia does not improve with levodopa. At the same time, evidence suggests that rasagiline is associated with significantly better odor-discrimination abilities in early-PD patients [49]. Smell impairment is more frequent in male patients [55] and is more severe in the PIGD subtype of PD [49]. Those were also findings in our study.

Over the past 10 years, several risk factors for PD have been identified in longitudinal studies [56]. Among the more important ones are age and gender [57]. In our study, alcohol drinking status was more common in the brainstem subtype, non-caffeine consumption status was less common in the limbic subtype, and non-smoking status was less common in the cortical subtype. Smoking and caffeine consumption have been consistently associated with a reduced risk of PD. However, the dual roles of positive and negative results from epidemiological studies on alcohol intake and PD risk have been reported [58]. Chronic alcohol use is associated with changes in brain structure and connectivity in the general population [59]. Some of the structures in the brainstem that are most affected by chronic alcohol consumption include raphe nuclei that are responsible for producing serotonin. This neurotransmitter plays a crucial role in mood regulation and sleep regulation. Chronic alcohol consumption can disrupt serotonin production and contribute to sleep disturbances, the most frequent in the brainstem subtype in our study [60]. Ma et al. [61] recently concluded that moderate and heavy drinkers had a significantly higher likelihood of having probable RBD, than non-drinkers. At least six large prospective epidemiological studies have firmly established a relationship between increased caffeine consumption and decreased risk of developing PD [62]. A recent systematic review concluded that caffeine consumption, especially in moderate quantities, may reduce the risk of dementia and cognitive decline, and ameliorate cognitive decline in cognitively impaired individuals [63]. Cho et al. [64] concluded that coffee consumption and tremor severity are inversely related in male patients with de novo PD, but not in female patients representing most of our study’s limbic subtype. In limbic subtype TD motor subtype dominated. Cigarette smoking exerts an undefined, biological, neuroprotective influence against the development of PD and AD [65]. Non-smoking status was significantly less common in the cortical subtype in our study. The observation that there were significantly more smokers among patients with the cognitive subtype is an interesting phenomenon; however, it is essential to note that this was an observational study, and we cannot establish a cause-and-effect relationship. It is possible that smokers had other common characteristics or lifestyle habits associated with cognitive decline. The observation could be coincidental.

Our study has limitations. First, the sample of patients may not be representative of a broader population of PD patients as our data were collected at one university center. We should expand our future work to a more ethnically diverse and larger sample, and a longitudinal study should be conducted. On the other hand, comparisons of individual motor and NMS subtypes of PB have been made on a sample of patients similar in size to ours [66,67,68,69].

Second, drug-naive patients would be ideal for investigating PD because there is a possibility that dopaminergic therapy may influence individual NMS. On the other hand, some studies have not found statistically significant differences between treated and untreated patients because other neurotransmitter systems are predominantly affected in NMS [66]. Since we already have a limited number of untreated patients for ethical reasons, the vast majority of the included patients were treated with symptomatic therapy. Following the example of previous studies [66], we calculated the L-dopa equivalent daily dose for all dopaminergic drugs.

Third, as PD is a heterogeneous disease, the most significant difficulty in phenotyping is the overlapping of specific subtypes, the instability of subtypes, and the potential change of NMS subtypes during disease progression (phenotypic conversion). The classifications are therefore an attempt to simplify the main subtypes of the disease, which most often overlap. Assessment of some NMS can be difficult in individual patients, mainly due to fluctuations in NMS, which may result from intrinsic compensatory mechanisms, drugs, or the natural course of the disease. For standardization, all patients were tested in the on phase.

Fourth, the K-means clustering algorithm applies a distance measurement to cluster the variables, which does not apply to categorical data types. Hence, only numerical variables were clustered. In addition, K-means clustering may not yield the same result with each run, because the resulting clusters depend on initial random assignments [18].

In the future, we intend to replicate both classification types in a second dataset. The problem is that the datasets must contain the same data types and values, requiring the same number of clusters. Paraclinical (radiological, neurophysiological, and genetic) biomarkers could also be included. Future studies with a rigorous design, standardized concerning the included variables, data processing, and clustering analysis technique, may advance the knowledge of PD subtypes [18].

## 5. Conclusions

Clinical subtyping allows for more accurate prognostication and improved treatment planning and enhances research into etiology, pathophysiology, and novel disease-modifying treatments. Our study found clear differences between individual NMS subtypes using both actuarial and data-driven approaches. The newly established algorithm has the potential to be used as a simplified tool for future clinical research of NMS subtypes of PD. However, external validation on a larger sample and prospective studies will be necessary.

## Figures and Tables

**Table 1:** Demographic and clinical characteristics of the overall study population based on a priori NMSS subtyping.

	Overall (n = 168) (100%)	Cortical (n = 38) (22.6%)	Limbic (n = 48) (28.6%)	Brainstem (n = 82) (48.8%)	*p*-Value	Adj. *p*-Value *	Post Hoc Statistical Analysis **
Gender (male)	100 (59.9%)	24 (63.2%)	20 (41.7%)	56 (68.3%)	0.010 ^χ2^	0.020	Limbic-Male/Femae
Age (years)	71.70 ± 9.57	77.08 ± 8.42	68.81 ± 8.88	70.89 ± 9.57	<0.001 ^KW^	0.055 (sig.)	Cortical-Limbic Cortical-Brainstem
Age at onset (years)	65.45 ± 10.18	72.21 ± 8.06	61.90 ± 9.55	64.40 ± 10.06	<0.001 ^KW^	0.014	Cortical-Limbic Cortical-Brainstem
Education ≥ 3. stage based on ISCED (%)	123 (73.2%)	18 (47.3%)	39 (81.2%)	66 (80.4%)	<0.001 ^KW^	0.028	Cortical-Limbic Cortical-Brainstem
Right-handed (%)	144 (85.7%)	23 (60.5%)	45 (93.8%)	76 (92.7%)	0.001 ^χ2^	0.018	Cortical-Left/Right
Disease duration > 10 years (%)	34 (20.2%)	3 (7.9%)	12 (25.0%)	19 (23.2%)	0.045 ^KW^	0.077	Cortical-Limbic
Family history	27 (16.1%)	11 (28.9%)	6 (12.5%)	10 (12.2%)	0.235 ^χ2^	0.294	
Side of onset—right (%)	92 (54.8%)	25 (65.8%)	23 (47.9%)	44 (53.7%)	0.245 ^χ2^	0.299	
	Motor subtype						
TD (%)	87 (51.8%)	2 (5.3%)	36 (75.0%)	49 (59.8%)	<0.001 ^χ2^	0.004	Cortical–TD/PIGD Limbic–TD/PIGD) Brainstem-PIGD
PIGD (%)	61 (36.3%)	33 (86.8%)	7 (14.6%)	21 (25.6%)			
Intermediate (%)	20 (11.9%)	3 (7.9%)	5 (10.4%)	12 (14.6%)			
No. of prodromes	2.2 ± 1.34	2.16 ± 1.26	2.17 ± 1.04	2.24 ± 1.53	0.947 ^KW^	0.947	
No. of NMS	6.88 ± 3.21	5.89 ± 2.15	7.25 ± 2.99	7.12 ± 3.66	0.154 ^KW^	0.207	
MoCA	25.77 ± 2.55	23.97 ± 1.94	26.50 ± 2.54	26.18 ± 2.43	<0.001 ^KW^	0.005	Cortical-Limbic Cortical-Brainstem
HAM-A	6.05 ± 5.32	2.58 ± 1.73	10.90 ± 5.07	4.83 ± 4.57	<0.001 ^KW^	0.005	All pairs
HAM-D	6.95 ± 5.61	3.11 ± 1.69	12.54 ± 5.50	5.46 ± 4.28	<0.001 ^KW^	0.005	All pairs
UPDRS III	37.37 ± 10.96	35.76 ± 9.11	39.85 ± 10.51	36.66 ± 11.85	0.130 ^KW^	0.183	
H&Y	2.45 ± 0.70	2.82 ± 0.51	2.33 ± 0.75	2.35 ± 0.69	<0.001 ^KW^	0.004	Cortical-Limbic Cortical-Brainstem
LED	725.00 ± 285.76	733.68 ± 283.27	685.62 ± 276.14	744.02 ± 293.50	0.522 ^KW^	0.563	
ESS	6.72 ± 4.24	6.79 ± 4.69	5.10 ± 2.68	7.63 ± 4.53	0.002 ^KW^	0.006	Limbic-Brainstem
FSS	31.81 ± 13.18	29.76 ± 10.74	35.67 ± 11.65	30.50 ± 14.64	0.023 ^KW^	0.044	Cortical-Limbic
RBDSQ	4.92 ± 2.65	4.39 ± 2.52	4.23 ± 2.15	5.57 ± 2.83	0.008 ^KW^	0.018	Limbic-Brainstem
SAS	11.35 ± 6.39	14.63 ± 5.75	10.35 ± 5.29	10.40 ± 6.81	0.001 ^KW^	0.003	Cortical-Limbic Cortical-Brainstem
NMSS	59.38 ± 36.94	48.92 ± 21.43	66.58 ± 34.87	60.00 ± 42.61	0.077 ^KW^	0.128	

* Benjamini–Hochberg method. ** Statistically significant pairs of post hoc analysis. ^χ2^ = χ^2^ test of independence, ^KW^ = Kruskal–Wallis test for independent samples. TD: tremor dominant; PIGD: postural instability gait disorder.

**Table 2:** Demographic and clinical characteristics of the overall study population based on cluster analysis.

	Cluster 1 (n = 37) (22.0%)	Cluster 2 (n = 35) (20.8%)	Cluster 3 (n = 38) (22.6%)	Cluster 4 (n = 46) (27.4%)	Cluster 5 (n = 12) (7.1%)	*p*-Value	Adj. *p*-Value *
Gender (male) (%)	21 (67.7%)	11 (33.3%)	21 (61.8%)	34 (65.3%)	13 (72.2%)	0.015 ^χ2^	0.025
Age at onset (years)	72.74 ± 8.71	62.42 ± 9.09	64.91 ± 10.05	64.10 ± 9.77	63.39 ± 11.03	<0.001 ^KW^	0.019
Disease duration > 10 years (%)	4 (12.9%)	7 (21.2%)	10 (29.4%)	5 (9.6%)	8 (44.4%)	0.003 ^KW^	0.006
Side of onset—right (%)	20 (64.5%)	18 (54.5%)	18 (52.9%)	29 (55.8%)	7 (38.9%)	0.543 ^χ2^	0.543
Motor subtype							
TD (%)	2 (6.5%)	28 (84.8%)	19 (55.9%)	29 (55.8%)	9 (50.0%)	<0.001 ^χ2^	0.003
PIGD (%)	28 (90.3%)	5 (15.2%)	9 (26.5%)	12 (23.1%)	7 (38.9%)		
Intermediate (%)	1 (3.2%)	0 (0.0%)	6 (17.6%)	11 (21.2%)	2 (11.1%)		
No. of NMS	6.58 ± 1.65	7.58 ± 2.54	8.03 ± 2.67	4.35 ± 2.66	11.28 ± 2.35	<0.001 ^KW^	0.005 (sig.)
MoCA	23.45 ± 1.75	26.55 ± 2.21	25.94 ± 2.41	27.10 ± 2.12	24.22 ± 2.32	<0.001 ^KW^	0.004
HAM-A	2.87 ± 2.13	12.33 ± 4.76	4.15 ± 2.23	3.02 ± 2.43	12.39 ± 5.17	<0.001 ^KW^	0.004
HAM-D	3.06 ± 1.18	14.27 ± 4.89	4.88 ± 2.27	3.83 ± 1.92	163.17 ± 5.44	<0.001 ^KW^	0.003
UPDRS III	36.29 ± 7.26	39.21 ± 6.80	38.85 ± 9.56	32.63 ± 11.93	46.72 ± 14.90	<0.001 ^KW^	0.003
H&Y	2.90 ± 0.40	2.27 ± 0.63	2.50 ± 0.66	2.04 ± 0.52	3.11 ± 0.83	<0.001 ^KW^	0.003
ESS	7.13 ± 4.77	5.18 ± 2.69	8.29 ± 5.24	5.52 ± 3.19	9.33 ± 4.23	<0.001 ^KW^	0.003
FSS	31.42 ± 9.91	35.12 ± 10.68	34.56 ± 14.14	24.77 ± 12.77	41.56 ± 12.35	<0.001 ^KW^	0.002
RBDSQ	4.90 ± 2.71	4.48 ± 2.32	5.85 ± 2.80	4.10 ± 2.07	6.39 ± 3.33	0.010 ^KW^	0.018
SAS	15.71 ± 5.62	10.85 ± 5.69	11.24 ± 5.56	7.77 ± 5.07	15.28 ± 7.51	<0.001 ^KW^	0.002
NMSS	56.90 ± 15.91	68.27 ± 21.48	64.47 ± 22.61	25.54 ± 13.04	135.44 ± 27.20	<0.001 ^KW^	0.005

* Benjamini–Hochberg method. ^χ2^ = χ^2^ test of independence, ^KW^ = Kruskal–Wallis test for independent samples. TD: tremor dominant; PIGD: postural instability gait disorder.

## Data Availability

The data presented in this study are available on request from the corresponding author. Requests to access the datasets should be directed to Timotej Petrijan, timotej.petrijan@gmail.com.

## Supplementary Materials

The following supporting information can be downloaded at: https://www.mdpi.com/article/10.3390/jcm12237434/s1, Table S1: Demographic and clinical characteristics of the overall study population based on a priori NMSS subtyping; Table S2: Frequencies of individual NMS in the overall study population and individual NMS subtype (a priori approach); Table S3: Frequencies of individual first NMS in the overall study population and individual NMS subtype (a priori approach); Table S4: Demographic and clinical characteristics of the overall study population based on cluster analysis.
