# Supplementary material for: Parkinson’s Disease Non-Motor Subtypes Classification in a Group of Slovenian Patients: Actuarial vs. Data-Driven Approach
Source: J Clin Med. 2023 Nov 30;12(23):7434. doi: 10.3390/jcm12237434 (PMC10707127; doi:10.3390/jcm12237434)
Supplement: Supplementary file 1 [file jcm-12-07434-s001.zip › jcm-2715781-supplementary.pdf]

Supplementary material

Table S1. Demographic and clinical characteristics of the overall study population based on a priori NMSS subtyping

	Overall (n= 168) (100%)	Cortical (n= 38) (22.6%)	Limbic (n=48) (28.6%)	Brainstem (n=82) (48.8%)	P-value	Adj. P-value (Benjamini-Hochberg)	Post hoc test (significant pairs)
**Gender (male)**	100 (59.9%)	24 (63.2%)	20 (41.7%)	56 (68.3%)	0.010 χ^2	0.020	**Limbic-M/F**
**Age (years)**	71.70 ± 9.57	77.08 ± 8.42	68.81 ± 8.88	70.89 ± 9.57	<0.001 KW	0.055 (sig.)	**Cortical-Limbic Cortical-Brainstem**
**Age at onset (years)**	65.45 ± 10.18	72.21 ± 8.06	61.90 ± 9.55	64.40 ± 10.06	<0.001 KW	0.014	**Cortical-Limbic Cortical-Brainstem**
**Age at onset 50-70 years (%)**	103 (61.3%)	12 (31.6%)	33 (68.8%)	58 (70.7%)	<0.001 KW	0.014	**Cortical-Limbic Cortical-Brainstem**
**Education ≥ 3. stage based on ISCED (%)**	123 (73.2%)	18 (47.3%)	39 (81.2%)	66 (80.4%)	<0.001 KW	0.028	**Cortical-Limbic Cortical-Brainstem**
**Right-handed (%)**	144 (85.7%)	23 (60.5%)	45 (93.8%)	76 (92.7%)	0.001 χ^2	0.018	**Cortical-Left/Right**
**Disease duration > 10 years (%)**	34 (20.2%)	3 (7.9%)	12 (25.0%)	19 (23.2%)	0.045 KW	0.077	**Cortical-Limbic**
**+ family history**	27 (16.1%)	11 (28.9%)	6 (12.5%)	10 (12.2%)	0.235 χ^2	0.294	
**Side of onset – right (%)**	92 (54.8%)	25 (65.8%)	23 (47.9%)	44 (53.7%)	0.245 χ^2	0.299	
			**Motor subtype:**				
**TD (%)**	87 (51.8%)	2 (5.3%)	36 (75.0%)	49 (59.8%)	<0.001 χ^2	0.004	**Cortical-TD/PIGD, Limbic-TD/PIGD Brainstem-PIGD**
**PIGD (%)**	61 (36.3%)	33 (86.8%)	7 (14.6%)	21 (25.6%)			
**Intermediate (%)**	20 (11.9%)	3 (7.9%)	5 (10.4%)	12 (14.6%)			
			**Risk factors**				
**Pesticides (%)**	53 (31.5%)	11 (28.9%)	11 (22.9%)	31 (37.8%)	0.196 χ^2	0.251	
**Solvents (%)**	28 (16.7%)	5 (13.2%)	7 (14.6%)	16 (19.5%)	0.617 χ^2	0.640	
**Rural environment (%)**	111 (66.1%)	20 (52.6%)	31 (64.6%)	60 (73.2%)	0.084 χ^2	0.136	
**Head injury (%)**	26 (15.5%)	10 (26.3%)	6 (12.5%)	10 (12.2%)	0.110 χ^2	0.168	

**Non-caffeine (%)**	56 (33.3%)	16 (42.1%)	7 (14.6%)	33 (40.2%)	0.005 ^{x2}	0.012	**Limbic-Y/N**
**Alcohol (%)**	53 (31.5%)	6 (15.8%)	10 (20.8%)	37 (45.1%)	0.002 ^{KW}	0.006	**Cortical-Brainstem Limbic-Brainstem**
**Non-smoking (%)**	104 (61.9%)	14 (36.8%)	31 (64.6%)	59 (72.0%)	0.003 ^{KW}	0.008	**Cortical-Limbic Cortical-Brainstem**
			**Prodromal NMS**				
**RBD (%)**	49 (29.2%)	14 (36.8%)	9 (18.8%)	26 (31.7%)	0.145 ^{x2}	0.199	
**Smell disorder (%)**	46 (27.4%)	14 (36.8%)	6 (12.5%)	26 (31.7%)	0.020 ^{x2}	0.039	**Limbic-Y/N**
**Constipation (%)**	51 (30.4%)	8 (21.1%)	9 (18.8%)	34 (41.5%)	0.009 ^{x2}	0.019	**Brainstem-Y/N**
**EDS (%)**	15 (8.9%)	3 (7.9%)	2 (4.2%)	10 (12.2%)	0.292 ^{x2}	0.349	
**Hypotension (%)**	23 (13.7%)	9 (23.7%)	4 (8.3%)	10 (12.2%)	0.104 ^{x2}	0.163	
**Sexual dysfunction (%)**	29 (17.3%)	5 (13.2%)	11 (22.9%)	13 (15.9%)	0.441 ^{x2}	0.505	
**Micturition dysfunction (%)**	17 (10.1%)	2 (5.3%)	5 (10.4%)	10 (12.2%)	0.502 ^{x2}	0.552	
**Depression (%)**	39 (23.2%)	3 (7.9%)	24 (50%)	12 (14.6%)	<0.001 ^{x2}	0.011	**All pairs**
**No. of prodromes**	2.2 ± 1.34	2.16 ± 1.26	2.17 ± 1.04	2.24 ± 1.53	0.947 ^{KW}	0.947	
**No. of NMS**	6.88 ± 3.21	5.89 ± 2.15	7.25 ± 2.99	7.12 ± 3.66	0.154 ^{KW}	0.207	
**MoCA**	25.77 ± 2.55	23.97 ± 1.94	26.50 ± 2.54	26.18 ± 2.43	<0.001 ^{KW}	0.005	**Cortical-Limbic Cortical-Brainstem**
**HAM-A**	6.05 ± 5.32	2.58 ± 1.73	10.90 ± 5.07	4.83 ± 4.57	<0.001 ^{KW}	0.005	**All pairs**
**HAM-D**	6.95 ± 5.61	3.11 ± 1.69	12.54 ± 5.50	5.46 ± 4.28	<0.001 ^{KW}	0.004	**All pairs**
**UPDRS III**	37.37 ± 10.96	35.76 ± 9.11	39.85 ± 10.51	36.66 ± 11.85	0.130 ^{KW}	0.183	
**H&Y**	2.45 ± 0.70	2.82 ± 0.51	2.33 ± 0.75	2.35 ± 0.69	<0.001 ^{KW}	0.004	**Cortical-Limbic Cortical-Brainstem**
**LED**	725.00 ± 285.76	733.68 ± 283.27	685.62 ± 276.14	744.02 ± 293.50	0.522 ^{KW}	0.563	
**ESS**	6.72 ± 4.24	6.79 ± 4.69	5.10 ± 2.68	7.63 ± 4.53	0.002 ^{KW}	0.006	**Limbic-Brainstem**
**FSS**	31.81 ± 13.18	29.76 ± 10.74	35.67 ± 11.65	30.50 ± 14.64	0.023 ^{KW}	0.044	**Cortical-Limbic**
**RBDSQ**	4.92 ± 2.65	4.39 ± 2.52	4.23 ± 2.15	5.57 ± 2.83	0.008 ^{KW}	0.018	**Limbic-Brainstem**

**SAS**	11.35 ± 6.39	14.63 ± 5.75	10.35 ± 5.29	10.40 ± 6.81	0.001 [KW]	0.003	**Cortical-Limbic Cortical-Brainstem**
**NMSS**	59.38 ± 36.94	48.92 ± 21.43	66.58 ± 34.87	60.00 ± 42.61	0.077 [KW]	0.128	

Table S2. Frequencies of individual NMS in the overall study population and individual NMS subtype (a priori approach)

NMS	Overall (n= 168) (100%)	Cortical (n= 38) (22.6%)	Limbic (n=48) (20.6%)	Brainstem (n=82) (48.8%)	P-value*	Adj. P-value (Benjamini-Hochberg)	Post hoc test (significant pairs)
**Salivation (%)**	38 (22.6%)	7 (18.4%)	9 (18.8%)	22 (26.8%)	0.444	0.498	
**Smell disorder (%)**	54 (32.1%)	16 (42.1%)	7 (14.6%)	31 (37.8%)	0.008	0.018	Limbic
**Dysphagia (%)**	36 (21.4%)	9 (23.7%)	8 (16.7%)	19 (23.2%)	0.635	0.647	
**Nausea/vomiting (%)**	40 (23.8%)	4 (10.5%)	10 (20.8%)	26 (31.7%)	0.034	0.060	
**Constipation (%)**	75 (44.6%)	10 (26.3%)	17 (35.4%)	48 (58.5%)	<0.001	0.009	Cortical, Brainstem
**Micturition dysfunction (%)**	81 (48.2%)	12 (31.6%)	19 (39.6%)	50 (61.0%)	0.004	0.010	Brainstem
**Sexual dysfunction (%)**	97 (57.7%)	19 (50.0%)	28 (58.3%)	50 (61.0%)	0.524	0.554	
**Dizziness (%)**	94 (56.0%)	20 (52.6%)	31 (64.6%)	43 (52.4%)	0.362	0.424	
**Sleep dysfunction (%)**	121 (72.0%)	24 (63.2%)	32 (66.7%)	65 (79.3%)	0.116	0.168	
**Legs swelling (%)**	40 (23.8%)	7 (18.4%)	16 (33.3%)	17 (20.7%)	0.179	0.234	
**Excessive sweating (%)**	48 (28.6%)	4 (10.5%)	12 (25.0%)	32 (39.0%)	0.005	0.012	Cortical, Brainstem
**Diplopia (%)**	21 (12.5%)	3 (7.9%)	2 (4.2%)	16 (19.5%)	0.024	0.044	Brainstem
**Pain (%)**	86 (51.2%)	15 (39.5%)	35 (72.9%)	36 (43.9%)	0.002	0.006	Limbic
**Depression (%)**	91 (54.2%)	14 (36.8%)	40 (83.3%)	37 (45.1%)	<0.001	0.008	Cortical, Limbic
**Anxiety (%)**	78 (46.4%)	7 (18.4%)	43 (89.6%)	28 (34.1%)	<0.001	0.007	Cortical, Limbic, Brainstem
**Psychosis (%)**	27 (16.1%)	2 (5.3%)	10 (20.8%)	15 (18.3%)	0.111	0.165	
**Cognitive impairment (%)**	102 (60.7%)	34 (89.5%)	26 (54.2%)	42 (51.2%)	<0.001	0.006	Cortical, Brainstem

Apathy (%)	53 (31.5%)	22 (57.9%)	11 (22.9%)	20 (24.4%)	<0.001	0.006	Cortical

*All hypotheses tested using χ^2 test of independence.

Table S3. Frequencies of individual first NMS in the overall study population and individual NMS subtype (a priori approach)

First NMS	Overall (n= 168) (100%)	Cortical (n= 38) (22.6%)	Limbic (n=48) (20.6%)	Brainstem (n=82) (48.8%)
Smell disorder (%)	32 (19.0%)	12 (31.6%)	3 (6.3%)	17 (20.7%)
Constipation (%)	33 (19.6%)	6 (15.8%)	5 (10.4%)	22 (26.8%)
Micturition dysfunction (%)	2 (1.2%)	0	1 (2.1%)	1 (1.2%)
Sexual dysfunction (%)	8 (4.8%)	2 (5.3%)	2 (4.2%)	4 (4.9%)
Dizziness (%)	9 (5.4%)	2 (5.3%)	4 (8.3%)	3 (3.7%)
Sleep dysfunction (%)	27 (16.1%)	8 (21.1%)	4 (8.3%)	15 (18.3%)
Legs swelling (%)	1 (0.6%)	0	0	1 (1.2%)
Excessive sweating (%)	10 (6.0%)	1 (2.6%)	2 (4.2%)	7 (8.5%)
Pain (%)	9 (5.4%)	2 (5.3%)	3 (6.3%)	4 (4.9%)
Depression (%)	19 (11.3%)	0	14 (29.2%)	5 (6.1%)
Anxiety (%)	13 (7.7%)	1 (2.6%)	10 (20.8%)	2 (2.4%)
Cognitive impairment (%)	4 (2.4%)	4 (10.5%)	0	0

Table S4. Demographic and clinical characteristics of the overall study population based on cluster analysis

	Overall (n= 168) (100%)	Cluster 1 (n=31) (18.5%)	Cluster 2 (n=33) (19.6%)	Cluster 3 (n=34) (20.2%)	Cluster 4 (n=52) (31.0%)	Cluster 5 (n=18) (10.7%)	P-value	Adj. P-value (Benjamini-Hochberg)
Gender (male) (%)	100 (59.9%)	21 (67.7%)	11 (33.3%)	21 (61.8%)	34 (65.3%)	13 (72.2%)	0.015 χ^2	0.025
Age (years)	71.70 ± 9.57	78.29 ± 8.60	69.33 ± 8.67	71.85 ± 10.29	68.58 ± 8.87	73.39 ± 7.94	<0.001 KW	0.038
Age at onset (years)	65.45 ± 10.18	72.74 ± 8.71	62.42 ± 9.09	64.91 ± 10.05	64.10 ± 9.77	63.39 ± 11.03	<0.001 KW	0.019
Age at onset 50-70 years (%)	103 (61.3%)	8 (25.8%)	24 (72.7%)	24 (70.6%)	37 (71.2%)	10 (55.6%)	<0.001 KW	0.013

**Education ≥ 3. stage based on ISCED (%)**	123 (73.2%)	14 (45.2%)	28 (84.8%)	26 (76.5%)	42 (80.8%)	13 (72.2%)	0.004 ^{KW}	0.008
**Right-handed (%)**	144 (85.7%)	20 (64.5%)	31 (93.9%)	31 (91.2%)	34 (65.4%)	17 (94.4%)	0.002 $^{\chi^2}$	0.004
**Disease duration > 10 years (%)**	34 (20.2%)	4 (12.9%)	7 (21.2%)	10 (29.4%)	5 (9.6%)	8 (44.4%)	0.003 ^{KW}	0.006
**+ family history**	27 (16.1%)	9 (29.0%)	4 (12.1%)	6 (17.6%)	6 (11.5%)	2 (11.1%)	0.449 $^{\chi^2}$	0.461
**Side of onset – right (%)**	92 (54.8%)	20 (64.5%)	18 (54.5%)	18 (52.9%)	29 (55.8%)	7 (38.9%)	0.543 $^{\chi^2}$	0.543
**Motor subtype:**								
**TD (%)**	87 (51.8%)	2 (6.5%)	28 (84.8%)	19 (55.9%)	29 (55.8%)	9 (50.0%)		
**PIGD (%)**	61 (36.3%)	28 (90.3%)	5 (15.2%)	9 (26.5%)	12 (23.1%)	7 (38.9%)	<0.001 $^{\chi^2}$	0.003
**Intermediate (%)**	20 (11.9%)	1 (3.2%)	0 (0.0%)	6 (17.6%)	11 (21.2%)	2 (11.1%)		
**Risk factors**								
**Pesticides (%)**	53 (31.5%)	9 (29.0%)	7 (21.2%)	13 (38.2%)	14 (26.9%)	10 (55.6%)	0.101 $^{\chi^2}$	0.132
**Solvents (%)**	28 (16.7%)	5 (16.1%)	1 (3.0%)	4 (11.8%)	13 (25.0%)	5 (27.8%)	0.056 $^{\chi^2}$	0.082
**Rural environment (%)**	111 (66.1%)	15 (48.4%)	21 (63.6%)	24 (70.6%)	37 (71.2%)	14 (77.8%)	0.170 $^{\chi^2}$	0.202
**Head injury (%)**	26 (15.5%)	10 (32.3%)	2 (6.1%)	4 (11.8%)	3 (5.8%)	7 (38.9%)	<0.001 $^{\chi^2}$	0.010
**Non-caffeine (%)**	56 (33.3%)	15 (48.4%)	29 (87.9%)	20 (58.8%)	36 (69.2%)	12 (66.7%)	0.014 $^{\chi^2}$	0.024
**Alcohol (%)**	53 (31.5%)	6 (19.4%)	8 (24.2%)	19 (55.9%)	13 (25.0%)	7 (38.9%)	0.019 $^{\chi^2}$	0.030
**Non-smoking (%)**	104 (61.9%)	13 (41.9%)	20 (60.6%)	24 (70.6%)	36 (69.2%)	11 (61.1%)	0.286 $^{\chi^2}$	0.320
**Prodromal NMS**								
**RBD (%)**	49 (29.2%)	11 (35.5%)	7 (21.2%)	9 (26.5%)	14 (26.9%)	8 (44.4%)	0.421 $^{\chi^2}$	0.444
**Smell disorder (%)**	46 (27.4%)	14 (45.2%)	4 (12.1%)	10 (29.4%)	12 (23.1%)	6 (33.3%)	0.046 $^{\chi^2}$	0.070
**Constipation (%)**	51 (30.4%)	5 (16.1%)	5 (15.2%)	20 (58.8%)	12 (23.1%)	9 (50.0%)	<0.001 $^{\chi^2}$	0.008
**EDS (%)**	15 (8.9%)	3 (9.7%)	0 (0.0%)	6 (17.6%)	5 (9.6%)	1 (5.6%)	0.152 $^{\chi^2}$	0.186

**Hypotension (%)**	23 (13.7%)	9 (29.0%)	4 (12.1%)	3 (8.8%)	6 (11.5%)	1 (5.6%)	0.087 $^{\chi 2}$	0.118
**Sexual dysfunction (%)**	29 (17.3%)	6 (19.4%)	6 (18.2%)	6 (17.6%)	5 (9.6%)	6 (33.3%)	0.240 $^{\chi 2}$	0.276
**Micturition dysfunction (%)**	17 (10.1%)	2 (6.5%)	1 (3.0%)	3 (8.8%)	8 (15.4%)	3 (16.7%)	0.311 $^{\chi 2}$	0.338
**Depression (%)**	39 (23.2%)	2 (6.5%)	17 (51.5%)	5 (14.7%)	9 (17.3%)	6 (33.3%)	<0.001 $^{\chi 2}$	0.006
**No. of prodromes**	2.2 ± 1.34	2.42 ± 1.23	2.06 ± 0.97	2.41 ± 1.74	1.85 ± 1.18	2.72 ± 1.49	0.106 [KW]	0.134
**No. of NMS**	6.88 ± 3.21	6.58 ± 1.65	7.58 ± 2.54	8.03 ± 2.67	4.35 ± 2.66	11.28 ± 2.35	<0.001 [KW]	0.005 (sig.)
**MoCA**	25.77 ± 2.55	23.45 ± 1.75	26.55 ± 2.21	25.94 ± 2.41	27.10 ± 2.12	24.22 ± 2.32	<0.001 [KW]	0.004
**HAM-A**	6.05 ± 5.32	2.87 ± 2.13	12.33 ± 4.76	4.15 ± 2.23	3.02 ± 2.43	12.39 ± 5.17	<0.001 [KW]	0.004
**HAM-D**	6.95 ± 5.61	3.06 ± 1.18	14.27 ± 4.89	4.88 ± 2.27	3.83 ± 1.92	163.17 ± 5.44	<0.001 [KW]	0.003
**UPDRS III**	37.37 ± 10.96	36.29 ± 7.26	39.21 ± 6.80	38.85 ± 9.56	32.63 ± 11.93	46.72 ± 14.90	<0.001 [KW]	0.003
**H&Y**	2.45 ± 0.70	2.90 ± 0.40	2.27 ± 0.63	2.50 ± 0.66	2.04 ± 0.52	3.11 ± 0.83	<0.001 [KW]	0.003
**LED**	725.00 ± 285.76	748.39 ± 296.56	673.18 ± 270.57	751.47 ± 234.74	682.88 ± 308.40	851.39 ± 294.87	0.077 [KW]	0.108
**ESS**	6.72 ± 4.24	7.13 ± 4.77	5.18 ± 2.69	8.29 ± 5.24	5.52 ± 3.19	9.33 ± 4.23	<0.001 [KW]	0.003
**FSS**	31.81 ± 13.18	31.42 ± 9.91	35.12 ± 10.68	34.56 ± 14.14	24.77 ± 12.77	41.56 ± 12.35	<0.001 [KW]	0.002
**RBDSQ**	4.92 ± 2.65	4.90 ± 2.71	4.48 ± 2.32	5.85 ± 2.80	4.10 ± 2.07	6.39 ± 3.33	0.010 [KW]	0.018
**SAS**	11.35 ± 6.39	15.71 ± 5.62	10.85 ± 5.69	11.24 ± 5.56	7.77 ± 5.07	15.28 ± 7.51	<0.001 [KW]	0.002
**NMSS**	59.38 ± 36.94	56.90 ± 15.91	68.27 ± 21.48	64.47 ± 22.61	25.54 ± 13.04	135.44 ± 27.20	<0.001 [KW]	0.005
